# Trypanosomatid parasites, translational research in a One Health, One World environment

**DOI:** 10.3389/fchem.2026.1815438

**Published:** 2026-05-15

**Authors:** Alicia Ponte-Sucre

**Affiliations:** 1 Department of Physiological Sciences, Chair of Human Physiology, Laboratory of Molecular Physiology, Institute of Experimental Medicine, Luis Razetti School, Universidad Central de Venezuela, Caracas, Venezuela; 2 Medical Mission Institute, Würzburg, Germany

**Keywords:** drug resistance, *Leishmania*, One Health, pharmacology, therapeutics, translational research, *Trypanosoma brucei*, *Trypanosoma cruzi*

## Abstract

Adequate policies to achieve universal access to high-quality diagnostics and therapeutics in the challenge represented by Trypanosomatidae-borne diseases remain essential to command surveillance as a successful tool and a way to delay the unavoidable surge of resistance toward the currently used drugs. In the case of leishmaniasis, even for uncomplicated cutaneous leishmaniasis, most patients in endemic regions are treated with systemic therapies due to reasons such as local protocol misalignment, limited access to new technologies, and lesions being located in anatomical areas other than the face or near the joints. These facts constitute a challenge for rural patients, especially in endemic areas, with limited access to medical facilities and compromising treatment adherence and clinical follow-up, thus increasing the evolution of treatment failure. In pharmacological-pharmaceutical terms, this means that there is an urgent need to minimize the rate of drug attrition and maximize the potential for success. For example, *in silico* computational chemistry data with no inclusion of *in vitro* or experimental data can produce more difficulties than advantages. The main reason is that the disparity between computational estimates and *in vitro*/*in vivo* studies may result in data that cycle within a never-ending loop, causing, despite enormous efforts, no great advance in producing novel compounds against parasites to treat the diseases. Thus, while considerable progress has been made in diagnosis, prevention, and treatment of Trypanosomatidae-transmitted diseases, significant challenges remain in the effort to develop new drugs in a One Health, One World environment. The consequence is high failure rates worldwide, a challenge we should all overcome to improve the outcome for a new drug portfolio.

## 1 Introduction

Trypanosomiases and leishmaniases, insect-borne parasitic diseases caused by protists from the clade Kinetoplastids, genera *Trypanosoma* and *Leishmania*, respectively, are widely distributed, more frequently in tropical and subtropical regions (Kasozi et al., 2022; Madeja and Schroeder, 2020; Steverding, 2008; 2010; WHO, 2021). These diseases have a huge medical and economic importance for the regions where they prevail as there are no vaccines to prevent these infections, and available drugs for their control are far from ideal due to host toxicity, limited access, and increasing rates of drug resistance.

### 1.1 Trypanosomiasis

#### 1.1.1 Human African trypanosomiasis

As wonderfully summarized by Hollingshead and Bermudez (2024), human African trypanosomiasis (HAT), or sleeping sickness, is caused by extracellular protozoans belonging to the genus *Trypanosoma*, species *brucei*. The disease spreads to susceptible hosts by the bite of the blood-sucking tsetse fly belonging to the genus Glossina. *Trypanosoma brucei gambiense* and *T. b. rhodesiense* are responsible for HAT (Swallow, 2000). Both subspecies share morphological features and life cycle patterns, but the pathological entities caused by them are unique, as are their epidemiological and clinical management patterns.⁠ *Trypanosoma* is a unicellular parasite. Its complex life cycle requires two hosts. In the human host, the parasite differentiates into a dividing, elongated, and “slender” form and a nonproliferating, short, and “stumpy” form (Funk et al., 2013; Mehlitz and Molyneux, 2019).

Slender trypanosomes invade body organs, including the central nervous system, through the bloodstream or lymphatics. In the target organs, slender parasites divide and reach a cell density that induces transformation into the stumpy (non-dividing) form through the stumpy induction factor, a secreted compound that induces parasite differentiation, thus balancing the parasite host levels over cell density. The transformed stumpy trypanosomes colonize the vector, which becomes infected after a blood meal. The life cycle continues, and proliferative procyclic stage parasites develop and colonize the vector’s gut, become the so-called nonproliferating mesocyclic stage, or travel to reach the proventriculus to become proliferative epimastigotes. These are directed toward the tsetse fly’s salivary glands, where, in addition to undergoing cell division, a daughter cell may attach to the salivary gland epithelium via its flagellum. This cell division is asymmetric. The long daughter cell dies, and the short daughter cell can either produce more attached, proliferating epimastigotes, or become the so-called non-multiplying, freely swimming, human-infective metacyclic trypomastigotes.

Issues related to HAT, such as etiological agent, infection stage, patient factors, transmission, treatment, and presence of contraindications, constitute rather complex matters. For example, children, women of reproductive age, or patients in the acute illness/reactivation phase must receive treatment both to limit morbidity and mortality and to prevent transmission. In the case of West African HAT, fexinidazole is the drug of choice for *T*. *brucei gambiense* treatment (Hollingshead and Bermudez, 2024).

Animal infections caused by *T. b. brucei*, *Trypanosoma vivax*, and *Trypanosoma congolense* are common in sub-Saharan Africa, constituting a major constraint for livestock production (Baker et al., 2013; Giordani et al., 2016; Kulohoma et al., 2020; Simo and Rayaisse, 2015), while *Trypanosoma evansi* affects a wide range of domestic and wild animals in both Africa and in Asia and South America (Okello et al., 2022; Richards et al., 2021). *T. evansi* is mechanically transmitted by biting flies. The non-vectorial *Trypanosoma equiperdum* is present in Asia, Africa, South America, Southern and Eastern Europe, Mexico, and Russia (Hamill et al., 2013), causing a venereal disease of horses. All these cases indicate the need for the implementation of progressive control pathways to make progress on the reduction, elimination, and eradication of human and animal disease (Diall et al., 2017).

#### 1.1.2 Chagas disease

*Trypanosoma cruzi* is the causative parasite for Chagas’ disease (CD), prevalent in South America, although also in countries of the northern hemisphere, especially due to human migrations (Chao et al., 2020). Chagas disease has been endemic in the Americas for millions of years. The complex disease epidemiology is shaped by countless conditions, with parasite genetics being an important one. The “discrete typing units” (DTUs) (TcI to TcVI plus TcBat) impose parasite genetic diversity. Additionally, challenges for understanding transmission dynamics and disease burden must embrace changes in the landscape of disease transmission and invasion of non-endemic settings (Chao et al., 2020; Pérez-Molina and Molina, 2018).

Blood-sucking bugs of the Triatominae (Hemiptera: *Reduviidae*) subfamily spread the disease, and more than 150 domestic and wild mammalian host species of many orders transmit the disease. This mainly occurs through a vector that simultaneously feeds on the blood of a susceptible mammal and evacuates feces containing infective parasite forms. These infectious parasites reach the blood system through skin lesions or mucous membranes. Transmission by house-infesting vectors has been reduced, and this has resulted in a decrease in the number of cases in the Americas. Estimates from the World Health Organization/Pan American Health Organization indicate that from the 1990s to 2010, case numbers went from ∼18 to 6–8 million infected people, and incidence fell from ∼200,000 to ∼40,000 new cases per year (WHO, 2021). However, Chagas disease is still endemic in Latin America.

Although discrepancies between these numbers and those from the Global Burden of Disease study are important (GBD 2023, 2025), what is clear is that Chagas disease control in the Americas remains an important issue. Approximately 90% of new infections go undiagnosed, with an estimated 70% of infected individuals unaware of their condition. Only approximately 1% of those infected and diagnosed at early stages of the disease receive specific treatment. If Chagas disease is left untreated, years later, chronic cardiac and digestive symptomatic forms appear with considerable morbidity, mortality, and overall burden. The prevalence of asymptomatic/oligosymptomatic features of most initial infections, along with limited awareness of the occurrence of Chagas disease, makes control of this ailment complex. A thorough description of this complexity is beautifully explained in the article by Cucunubá et al. (2024), including the challenging issue represented by Chagas disease diagnosis.

### 1.2 Leishmaniasis

This dreadful infectious disease caused by the unicellular parasite *Leishmania*, distributed throughout all continents, is mainly present in the tropics, subtropics, and southern Europe. It is transmitted to humans by phlebotomine (Old World) and Lutzomyia (New World) female sand flies (WHO, 2021). It is considered the third most important vector-borne parasitic disease worldwide, after lymphatic filariasis and malaria. Due to its widespread occurrence, it is additionally associated with conditions affecting neglected populations, such as malnutrition, poor domestic sanitary conditions, a weak immune system, and scarce resources. According to the WHO, more than 1 billion people live in endemic areas for leishmaniasis and remain at risk of infection, accounting for approximately one million new cases of cutaneous and 30,000 new cases of visceral leishmaniasis each year. The increase observed in the geographical distribution of leishmaniasis is also associated with human migration, conflicts, and climate change.

Many species of *Leishmania* cause both visceral (VL) and tegumentary leishmaniasis (TL) (Alunda et al., 2024). VL, also known as kala-azar, is caused mainly by *L*. (*L.*)* donovani* in East Africa, India, China, Iraq, and Nepal, and by *L*. (*L.*)* infantum* (previously called *L*. (*L.*) *chagasi* in Latin America) in Europe, North Africa, and Latin America. Species that produce diverse forms of TL include members of the *Leishmania* and *Viannia* subgenera. This has been thoroughly described by Scorza et al. (2017) for each type of TL. Cutaneous leishmaniasis (CL) includes species that, in the Old World, are *L.* (*L.*) *major*, *L.* (*L.*) *tropica*, *L.* (*L.*) *aethiopica*, *L.* (*L.*) *infantum*, and, in rare cases, *L.* (*L.*) *donovani*, and for the New World are *L.* (*L.*) *mexicana*, *L.* (*L.*) *amazonensis*, *L.* (*V.*) *braziliensis*, *L.* (*V.*) *panamensis*, *L.* (*V.*) *guyanensis*, and *L.* (*V.*) *peruviana*. CL is the commonest form of leishmaniasis, with a diversity of symptoms in the skin, ranging from simple lesions and ulcers to disfiguring lesions that leave lifelong scars in exposed parts of the body. The areas most affected are the Mediterranean basin, the Middle East, Central Asia, and the Americas. Mucocutaneous leishmaniasis (MCL) and disseminated cutaneous leishmaniasis (DCL) are commonly seen in the New World. The main invading species are *L.* (*V.*) *braziliensis*, *L.* (*V.*) *panamanensis*, *L.* (*V.*) *guyanensis*, and *L.* (*L.*) *amazonensis.* MCL is an ailment that affects the mucous membranes of the nose, mouth, and throat and promotes their destruction, being the most disabling form of the disease. Most cases occur in Brazil, Bolivia, Ethiopia, and Peru. Post-kala-azar dermal leishmaniasis (PKDL) occurs in patients cured of VL and is characterized by the development of rashes of macular, papular, or nodular eruptions usually on the face, upper arms, and other parts of the body skin, a few months or years after the cure of VL. PKDL is more frequently observed in the Old World and is associated with *L.*(*L.*) *donovani* as the main species.

Finally, zoonotic leishmaniasis, caused principally by *L*. (*L.*) *infantum*, affects the canine population in the Mediterranean Basin and in South America, particularly in Brazil. Infected dogs are considered one of the main reservoirs of human infections in these regions (Mendonça et al., 2025).

## 2 Trypanosomatidae peculiar features

Economic and social interests guide the need to control these diseases; however, the results obtained have always fallen short of expectations. These are complex ailments, transmitted by vector species with different ecological and ethological characteristics. Vertebrate hosts may be diverse and function as reservoirs. Environmental control is normally not successful. Usually, the diseases caused by these parasites are chronic and can present an asymptomatic course that makes even humans potential transmitters. Parasites may have different pathogenicity and immunogenicity, including a spectrum of how the target species react immunologically speaking. Human vaccine development has been elusive for both *Trypanosoma* and *Leishmania*, and the marketed vaccines against zoonotic *L*. (*L.*) *infantum* in dogs have serious drawbacks (Alunda, 2024). Finally, although leishmaniasis, CD, and HAT are primarily transmitted by various insect vectors, Chagas disease can also be transmitted congenitally, through contaminated blood, and through contaminated food and drinks (De Rycker et al., 2023).

Due to the complexity already explained, it is clear that strategies to control these neglected tropical diseases (NTDs) in the long term must rely on approaches that come from different fields, including education of affected and at-risk human populations, and limitation of vector spread through sanitation, adequate housing, and nearby medical and veterinary services. Accessible and affordable antiparasitic drugs must have a tolerable efficacy–toxicity balance, be easy to administer, and have a rapid onset of action. All strategies must take into consideration that parasites and hosts are constantly evolving to adapt to new conditions and thus that the probability of therapeutic failure emergence and of drug-resistance selection may be a natural consequence, especially if poor surveillance programs are used. Therefore, a panorama is created in which the growing resistance to chemotherapy reduces the therapeutic arsenal to treat Trypanosomatidae infections, as has been thoroughly described for the first-line drugs against *Trypanosoma* and *Leishmania*, due to the emergence of drug resistance (Alunda, 2024).

The presented reality has stimulated research efforts to identify new drug targets in HAT-causing *Trypanosoma*, *T. cruzi*, and *Leishmania* (de Menezes et al., 2015). These facts are beautifully discussed in the special volume of *Microorganisms* edited by Alunda (2024). There, authors discuss both molecular targets and the research tools that could help to identify and validate essential genes and, hence, guide the identification of new chemotherapeutic targets, including the usefulness of repurposing, combination, and new presentations of “old drugs” and the consequences this may have in the reduction of treatment failures and the emergence of resistance (Akhoundi et al., 2017; Alunda, 2024; Jamabo et al., 2023; Salomão and De Castro, 2017).

## 3 Globalization of zoonoses, including those produced by Trypanosomatidae

Humans are curious and explore and colonize new geographical areas. When they do so, the spread of microorganisms, including parasites, occurs. Voluntary migration (emigration, immigration, external and internal migration, and labor migration), along with forced migration (in previous times, the transatlantic slave trade) and displacement and relocation of people caused by different forms of violence, constitute the main causes for the dispersal of parasites accompanying humans throughout the world. In addition, trade and tourism are effective modes of expansion of the geographical areas where microorganisms live around the world, even invading non-endemic areas (Lewis et al., 2023).

It is well known that people from Asia moved, via Alaska and North America, to Latin America (LA) many centuries ago and thus constitute the ancestors of the autochthonous LA-people. It is also true that Latin America is a continent “rediscovered” a few centuries ago and, thus, Latin American people were in a way “isolated” before that moment. This fact thus transformed LA into a natural laboratory of native indigenous people and of their relationship with nature, and how this transduced into traditional knowledge and the interconnections developed through the history of LA and what it is now. Latin American autochthonous populations developed complex cultures, including the Aztec, Inca, and Mayan, along with those near the Amazon, the Caribbean, and the Guarani in the southern parts of South America. Thus, LA is a melting pot of cultures. The arrival of European explorers at the end of the 15th century and later on of African enslaved people, animals, and plant species not autochthonous to the continent constituted additional elements that shaped the health and life of native Latin American species, including humans. The result was an adaptive and interdependent human-nature relationship typical for LA (Seymour, 2016). This suggests that the health concept, especially for this 21st century, should incorporate concepts from early times of native LA, including the balance that existed between the natural environment and indigenous life, culture, and history (Pettan-Brewer et al., 2021; Vergara, 2018). Additionally, globalization of zoonoses constitutes a challenge due to greater connectivity between regions and changes in land use, including deforestation. Climate changes, evidenced in the current global warming, are certainly co-protagonists of the increase in geographical distribution of microorganisms, including parasites causing diseases, along with their human hosts (Lebarbenchon et al., 2008; Rosenthal, 2009; Steverding, 2020).

## 4 *Leishmania* and trypanosomes in Latin America: autochthonous or migrant parasites?

The classical notion about the genus *Trypanosoma* was that it is divided into the species *T. cruzi* in the Americas and *T. brucei*, *congolense*, *evansi, equiperdum*, *rhodesiense*, *vivax*, etc., in Africa. However, history may reveal a different reality. For example, *T. evansi*, *T. equiperdum*, and *T. vivax* are parasites that evolved approximately 35 million years ago in Africa (Steverding, 2020), and they affect mainly equids, camelids, and livestock animals in general. Because these animals constituted the main means of mobilization during the conquest of territories by humans, they were the main vehicles by which the parasites traveled from one place to another and from one continent to the next (Claes et al., 2005; Desquesnes et al., 2009; Milocco et al., 2013).

Interestingly, these three species of trypanosomes have different modes of transmission, each being very sophisticated. *T. evansi*, which is the etiological agent of Surra, is usually mechanically transmitted through the bite of tabanids and stomoxes and in America via the common vampire bat, *Desmodus rotundus* (Desquesnes et al., 2008), which not only acts as a biological vector but also as a host and reservoir for the parasite. Meanwhile, *T. equiperdum*, the etiological agent of dourine in horses, is transmitted directly from one animal to another during sexual contact (Brun et al., 1998). *T. vivax*, responsible for nagana disease in cattle, is cyclically transmitted by tsetse flies (in Africa) and mechanically by biting flies such as tabanids in America (Desquesnes and Dia, 2004).

*T. evansi* is thought to have been introduced into different places outside Africa due to the military campaigns conducted by ancient Egyptians in the Middle East, aiming to reach Asia and India in the eighth century (Desquesnes et al., 2013). Its initial introduction into Colombia, South America, occurred due to the Spanish conquistadores in the sixteenth century and in the nineteenth century in Brazil (Desquesnes and Dia, 2004). Because horses were the main form of land transportation for many centuries, *T. equiperdum* may have spread worldwide due to the horse trade (Desquesnes and Dia, 2004). Systematic screening and control helped to eradicate *T. equiperdum* from North America and Western Europe, especially after the Second World War (Claes et al., 2005; Desquesnes and Dia, 2004). The arrival of *T. vivax* into Latin America coincided with the arrival of zebu cattle from Senegal to French Guyana, Guadeloupe, and Martinique in 1830, although it probably arrived before, as early as 1733, on cattle arriving from Africa, directly from or through the Caribbean islands (Maillard and Maillard, 1998).

Obvious reasoning indicates that *T. brucei* should have traveled over the Atlantic Ocean with the African enslaved people (Steverding, 2014), but it never became established in America because tsetse flies, the insect vectors transmitting the disease, do not have America as one of their ecological niches.

In the case of *T. cruzi*, the history goes backward. That is, *T. cruzi* evolved from a bat parasite; it is younger than African trypanosomes. This event seems to have happened approximately 10 million years ago in South America (Steverding, 2014). These parasites are very “creative.” In the case of *T. cruzi*, especially, the feces of triatomine bugs (kissing bugs or conenose bugs) constitute the main form of transmission, not excluding blood transfusion, breastfeeding, congenital transmission, organ transplantation, and, last but not least, ingestion of contaminated food and drinks. At present, oral transmission has become the primary or one of the main routes of Chagas disease transmission, particularly in Brazil, but also very often present in different countries from Latin America, such as Bolivia, Colombia, French Guyana, and Venezuela (López-García and Gilabert, 2023; Noya et al., 2015). These additional means of transmission make *T. cruzi* a potential global parasite, as South Americans move throughout the world by migration, although the triatomine vector species are exclusively located in tropical areas of LA (Dujardin et al., 2015). A note of caution should be mentioned because enzootic infection of wild animals and autochthonous infections of humans have been reported from some southern states of the United States (Cantey et al., 2012).

Regarding *Leishmania*, it is interesting to learn that a Trypanosomatidae line of monogenetic insect parasites appears to have diverged and become *Leishmania* parasites (Fernandes et al., 1997). Although the evolutionary pressures experienced by *Leishmania* parasites might be understood if their phylogeny were thoroughly comprehended, knowledge of phylogeny alone will not be sufficient, as aspects of their biology are also needed. However, the existing data supported by small subunit ribosomal RNAs indicates that approximately 40–65 million years ago, *Leishmania* evolved from monogenetic insect flagellates (Akhoundi et al., 2016; Shaw, 1997).

All species, except *L.* (*L.*) *chagasi*, seem to have been inhabitants of different continents long before modern humans evolved (Tuon et al., 2008). *L.* (*L.*) *chagasi* is identical to *L.* (*L.*) *infantum*; however, their common origin has been widely debated (Chocholová et al., 2008), and it has been reported that it has been introduced into America in post-Columbian times (Bayesian phylogenetic analysis demonstrating strong similarities between the *L. chagasi* clusters and the Portuguese *L.* (*L.*) *infantum* clade), suggesting that the parasite migrated with the conquistadores and their dogs to South America approximately 500 years ago (Steverding, 2017; 2020). These considerations led to *L.* (*L.*) *chagasi* recently being renamed worldwide as *L*. (*L.) infantum.*

However, the arrival of *L.* (*L.*) *infantum* into the Americas cannot be taken as an accident. It constitutes a special case that brings together migration, evolution, and adaptation (fitness) of this unique parasite into an environment far from its original home. It illustrates unique issues related to host/pathogen co-evolution that, due to its public health consequences, constitutes a “school” to learn changes in disease manifestation associated with the novel natural selection and bottlenecks that represent a challenge for parasite survival and the natural genetic drift that should occur to guarantee successful evolutionary changes. This is especially so if additional vector species are involved in the spread of the parasite. A wonderful insight into this theme can be found in the work published by Boité et al. (2019).

A take-home lesson is that the initiation of the globalization of leishmaniasis and trypanosomiasis has been enabled by the zoonotic characteristics of these pathologies. Asymptomatic carriers, together with the spread of the vectors of these parasites, have been key factors in their spread all over the world.

## 5 Drugs for leishmaniasis and trypanosomiasis: options, challenges, and a new way of approaching this challenge

Upon exploring the stock of medications against these diseases, one can envision that for human African trypanosomiasis (HAT), the situation has improved. However, it should be realized that the drastic decrease in HAT is mainly due to improved surveillance (Mumba Ngoyi et al., 2014). This is not the case for leishmaniasis and especially for Chagas disease (CD), which urgently needs new treatments. Challenges in developing new clinical candidates are enormous, and inspiring solutions should be developed to overcome them.

### 5.1 Trypanosomiasis

#### 5.1.1 Human African trypanosomiasis

The current drug treatment for HAT (see Table 1) has been used for decades. As mentioned, HAT incidence has decreased dramatically and to historic low levels, but as long as one case exists, we should continue in the goal to control and prevent this disease thoroughly. The patients are classified as Stage 1 (absence of trypanosomes and white blood cell counts ≤5/µL) or Stage 2 (presence of trypanosomes and white blood cell counts ≥20/µL), as well as the intermediate stage for *T. b. gambiense* HAT that lies within these controversial thresholds (see Table 1).

**TABLE 1:** Current drugs used against Stage 1 and Stage 2 human African trypanosomiasis.

Stage and form of HAT	Parenteral first-line therapy	Parenteral second-line treatments/ Therapeutic failure treatments
Gambian HAT (*T. gambiense*): West and central Africa, 90% of cases, mostly from the Democratic Republic of Congo		
Stage 1	Pentamidine isethiocyanate	Eflornithine/melarsoprol
Stage 2	Combined nifurtimox/eflornithine therapy (NECT)	
Rhodesian HAT (*T. rhodesiense*): East Africa, 10% of cases, mostly from Uganda and Tanzania		
Stage 1	Suramin	Melarsorpol
Stage 2	Melarsoprol	

Modified from Bouteille and Buguet (2012) and Gao et al. (2020).

Fexinidazole is an effective compound for first- and second-stage disease except in highly advanced stages. In addition, pentamidine may be prescribed, especially for younger patients and people with fexinidazole contraindications or who do not meet treatment criteria. Follow-up, including blood and cerebrospinal fluid (CSF) reexamination, is a must in patients treated with fexinidazole for up to 24 months to assess disease relapse. Pentamidine is ineffective in second-stage disease; it does not cross the blood-brain barrier. Details may be found in the article written by Hollingshead and Bermudez (2024). Stage 1 patients can be treated on site with pentamidine or suramin. In the case of West Africa, first-stage *T. brucei rhodesiense*-infected patients with contraindications to fexinidazole could also be treated with suramin. Of note, due to the risk of severe drug-induced allergies, suramin use is usually hampered in West and Central Africa.

For second-stage disease, the situation may be complicated depending on the white blood count in cerebrospinal fluid. Details may be found in the article written by Hollingshead and Bermudez (2024). However, still, eflornithine is more efficient in reducing mortality and less toxic than melarsoprol and is the preferred medication for second-stage disease. Even more, the combination of nifurtimox and eflornithine reduces both dosage and cost of therapy. Fexinidazole is additionally effective in attacking *T. brucei rhodesiense* disease. If patients cannot be treated with fexinidazole, melarsoprol may be used.

This is not the case for Stage 2 patients, who must be treated in specialized facilities and with drugs that are both highly toxic and/or require complex administration procedures; that is, combination therapy with melarsoprol, eflornithine, or nifurtimox–eflornithine. Even more, suramin and melarsoprol constitute the unique treatment active against Rhodesian HAT.

#### 5.1.2 Chagas disease

Since the first description of Chagas disease at the beginning of the 20th century, several compounds, such as arsenic, fuchsin, bismuth, antihistamines, amphotericin B, antibiotics, and others, have been used for treatment. This has been very well summarized by Coura and De Castro (2002) and García Huertas and Cardona-Castro (2021). Currently, only two drugs are available for its treatment, nifurtimox (Lampit) and benznidazole (Rochagan) (Sánchez-Sancho et al., 2010). These medications are indicated for acute and congenital infections, chronically infected children, and cases of disease reactivation. In fertile women with chronic asymptomatic infections, benznidazole is recommended. It seems to reduce the risk of vertical transmission, but its benefits are not that obvious for adults with indeterminate chronic disease. Benznidazole is well tolerated, but side effects may trigger an interruption of treatment in up to 35% of chronically infected adult patients.

Their mechanism of action is still unknown, although they act as pro-drugs, activated within the parasite by a mitochondrial NADH-dependent type-I nitroreductase (Mejia et al., 2012; Wilkinson et al., 2008). This metabolism results in the production of free radical intermediates and electrophilic metabolites, for example, the cytotoxic metabolite glyoxal, which prevents the formation of new DNA chains (Hall and Wilkinson, 2012); additionally, unsaturated open-chain nitriles are produced due to nifurtimox reduction that triggers their reaction with toxic cellular components for the parasite (Hall et al., 2011). Nifurtimox and benznidazole function mainly in early infection phases (Hall et al., 2011; 2012), being extremely toxic (adverse effects include anorexia, nausea, vomiting, headache, central nervous system depression or maniacal symptoms, seizures, vertigo, paresthesia, peripheral polyneuropathies, and dermatitis), a situation that impacts therapy continuation in a high percentage of patients (Coura, 2009).

We should stress that an effective treatment for chronic Chagas disease must kill extra- and intracellular parasites, including those that are either dormant or in cryptic forms that, due to their functional characteristics or hidden place, are protected from the trypanocidal action of medications and therefore resistant to their action (Sánchez-Valdez et al., 2018). Chagas disease treatment includes the use of specific anti-*T. cruzi* drugs but, more importantly, the adequate treatment and management of heart and digestive system chronic accompanying diseases (De Rycker et al., 2023). Symptomatic forms need additional specialized care for heart failure, arrhythmias, and thromboembolism, and chronic digestive forms should receive special care that may span from a special diet to surgery. For example, the treatment of megaoesophagus aims at relieving dysphagia by facilitating the passage of food through the lower esophageal sphincter through surgery.

The WHO recommends this specific anti-trypanosoma treatment in all chronic-phase *Trypanosoma cruzi*-infected individuals. However, this new paradigm is considered controversial in current medical practice, meaning that chronic Chagas patients receive only palliative treatment for adult Chagas patients with dilated cardiomyopathy. Etiological treatment should now be mandatory for all adult chronic Chagas disease patients, a practice that should be introduced and consolidated within clinical practice to further prevent secondary ailments associated with this complex disease (Viotti et al., 2013).

### 5.2 Leishmaniasis

The main medications used for many decades to treat VL and TL belong to the antimonials, with the pentavalent antimonial salts, sodium stibogluconate and meglumine antimoniate, being the ones used (de Menezes et al., 2015; Sundar et al., 2024). However, many challenges exist for their administration because daily parenteral administration for many weeks makes the situation difficult. Additionally, the side effects of its administration make adherence to the therapy rather complicated. Declining efficacy and a low safety profile, especially for VL, are major drawbacks (see Table 2).

**TABLE 2:** Current drugs used against visceral leishmaniasis (VL), tegumentary leishmaniasis (TL), cutaneous leishmaniasis (CL), mucocutaneous leishmaniasis (MCL), and post-kala-azar dermal leishmaniasis (PKDL).

Drug	Topical/intralesional	Systemic	Oral	Introduction year	VL	PKDL	TL (CL, MCL)
Pentavalent antimonials	X	X	​	1937–1945	X	X	X
Amphotericin B	​	X	​	1959	X	X	​
Miltefosine	​	​	X	2002	X	​	X
Paromomycin	X	X	​	2006	X	​	X*
Pentamidine	​	X	​	1973	X	​	X
Azoles	X	​	X	1980	​	​	X
Allopurinol	​	​	​	1980	X	​	​

* Only for CL; modified from Olias-Molero et al. (2021) and Pinheiro and de Souza (2022).

Additionally, as we will discuss later, the development of parasites that are resistant to these compounds has forced the use of repurposed compounds, either antimicrobials (pentamidine and paromomycin), antifungals (amphotericin B, fluconazole, and ketoconazole), or, more recently, antitumor (miltefosine). This means that the brochure of compounds is associated with serious limitations, mentioning only two: toxicity and prolonged administration in a hospital or care unit environment. Finally, there have been reports of increasing rates of therapeutic failure, which develops due to the selection of resistant parasites to some of these compounds in clinical settings (Alunda, 2024). Treatment of TL, especially CL, is very particular, especially depending on the infecting species, how severe the lesions are, and the possibility of turning into MCL (Zhang et al., 2025).

Amphotericin B (AmB) deoxycholate began to be used due to the widespread resistance to antimonials, initially in India. Again, issues related to toxicity (such as nephrotoxicity, hypokalemia, and cardiotoxicity), along with infusion reactions, imply frequent laboratory monitoring and even the need for long hospitalization, which makes the use of this compound rather complicated. For TL, the liposomal formulation of amphotericin B has been successful in cases produced by Old World *Leishmania* with intravenous or intralesional injections (Sundar et al., 2024).

Miltefosine has constituted a unique oral agent against VL in use since 2002. Cure rates in India, Nepal, and Bangladesh are good (Monge-Maillo and López-Velez, 2015). However, increasing rates of clinical failures have been reported, and cases of drug resistance have appeared. In East Africa, its efficacy has been considered moderate, but in the Mediterranean countries and Latin America, the situation has not been completely successful. Added to these issues, potential teratogenicity, the need for contraception for nearly 6 months, and significant noncompliance owing to a 28-day treatment regimen, have impaired the spread of its use and promoted the change in therapy toward liposomal amphotericin B, especially in the Indian Subcontinent. However, there are difficulties inherent to this drug, including transport, administration (refrigeration and intravenous), and, last but not least, cost. This situation expands for patients coinfected with VL and human immunodeficiency virus (HIV). It has been used for canine VL in Brazil (Dos Santos Nogueira et al., 2019).

Much conflicting data surround the use of miltefosine for treating TL, especially CL, caused by some New World and Old World species, as well as in Iran, Colombia, and even Brazil. In the Old World, CL is most often caused by *L.* (*L.*) *major* and *L.* (*L.*) *tropica*. CL lesions heal spontaneously, and the use of intralesional antimonials and the use of an ointment containing pentamidine and methyl benzethonium chloride has been successful, as well as cryo- and thermotherapy. Data regarding New World CL are contradictory, with results that depend mostly on the infecting species (Sundar et al., 2024).

The azoles are mainly used for TL, especially CL, with differing results when comparing systemic vs. topical application and depending on the infecting parasite. In both Old and New World CL, paromomycin, allopurinol, and azoles have been used either alone or in combination with antimonials. In these cases, success can still vary. Finally, antimonials remain the most commonly used treatment for MCL, with moderate effectiveness, and there is existing evidence that mentions that use of miltefosine for MCL may be comparable to that of antimonials (Sundar et al., 2024).

**FIGURE 1:**
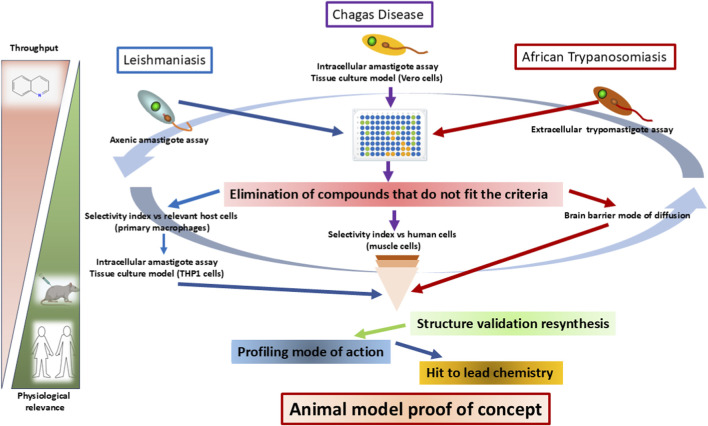
Experimental critical paths that require attention to be successful in the search for potential candidates against leishmaniasis, Chagas disease, and human African trypanosomiasis: (1) identify compounds that are active in axenic and intracellular models and extracellular models in the case of human African trypanosomiasis. (2) High-throughput screening. (3) Non-selective compounds compared to human cells are eliminated. (4) Validation through structure and purity determination and/or resynthesis. (5) Active compounds are analyzed through known modes of action. (6) Validated hits progress to hit-to-lead chemistry to achieve proof-of-concept efficacy in available disease animal model experiments. The image of the quinoline ring was obtained from https://pubchem.ncbi.nlm.nih.gov/compound/Quinoline#section=Structures. The mouse image was obtained from https://www.assaygenie.com/in-vitro-vs-in-vivo-complete-comparison-selection-guide-research-methods/?srsltid=AfmBOooWcmSosKkvRelXhrmagaD6QodcMctpnj6zZaKipISd7E4ohzEU. The image of the humans was obtained from https://www.educima.com/dibujo-para-colorear-hombre-y-mujer-i21995.html.

In both CD and leishmaniasis, the prevailing model to develop new drugs has poor efficiency, and there is a high failure (attrition) rate of potential drugs. This is partially due to the unfortunate predictive value of preclinical research, lack of common goals of collaborative research, fragmented efforts, use of inadequate surrogate models, especially for *in vivo* trials, and shortcomings of target product profiles (Olias Molero et al., 2021). Figure 1 illustrates the experimental critical paths that require attention to be successful in the search for potential candidates (De Rycker et al., 2023).

## 6 Challenges and new ways of approaching the challenge

Although the situation for HAT treatment has improved (Dickie et al., 2020; Kennedy, 2013; 2019), especially due to the development of new treatments, and that VL has seen some new compounds with novel modes of action in early clinical development, CL, MCL and PKDL face an urgent need for new drugs (Zhang et al., 2025), and for achieving the WHO’s road map for NTDs (WHO, 2020; 2021), which aims to fulfil the United Nations Sustainable Development Goals (Sánchez-Valdez et al., 2018).

Among the highest challenges that impose hurdles in the development of new drugs against these diseases, we must mention (a) the existence of “persister” forms in *T. cruzi* CD (Bustamante et al., 2020; Sánchez Valdez et al., 2018) and *Leishmania* spp. (Barret et al., 2019; Pinheiro and de Souza, 2022), (b) the unknown percentage of infected people with no symptoms who are difficult to identify, are potential reservoirs of infection in the case of leishmaniasis, and are classified as indeterminate, who may, in the future, develop clinical symptoms of CD, and (c) the action of animals as reservoirs of infection (De Rycker et al., 2023).

Although inequities in global research and development have been well known for decades, it was only in 1996 that the Commission on Health Research for Development highlighted them by publishing a report (Bryant and Harrison, 1996). The commission stated that 90% of the global disease burden is accounted for by the world’s poorest nations and that only 10% of research was geared toward treating the conditions that caused such significant morbidity and mortality (O’Neale Roach, 2000). Since then, the international community has made strong efforts to address the disparity existing between healthcare systems and search for solutions to the treatment of NTDs. Even pharmaceutical companies aligned with the WHO’s NTDs Roadmap 2030 to “respond to NTDs over the next decade” (WHO, 2020; 2021).

For example, Sanofi has been working with the Drugs for Neglected Diseases initiative (DNDi) on a new treatment for HAT, acoziborole, that, once approved, will be distributed at no cost to patients or governments (DNDi, 2020). This compound was identified by researchers from the University of California at San Francisco and Sanofi biotech partners at Anacor and Scynexis. It was selected for lead optimization and, after receiving a new status in 2009 as a chemical entity from DNDi, entered clinical development with the aim of starting a new approach to HAT treatment. One of the main issues is that toxicity and complexity could be avoided because it does not include arsenic, as is the case with melarsoprol (Barrett, 2025). Additionally, its use prevents treatment with less toxic compounds such as the combination therapy nifurtimox–eflornithine (NECT), for which distribution to remote regions constitutes a logistical challenge and needs trained nursing staff for administration and hospitalization for patients.

DNDi and Sanofi, working together, were pioneers on the first all-oral treatment for HAT, fexinidazole. This approach avoids patient systematic hospitalization and lumbar puncture associated with NECT. In 2018, market authorization was granted for a 10-day treatment of patients in the Democratic Republic of Congo (DRC) after the positive scientific opinion received from the European Medicines Agency (DNDI, 2018). Still, for those living in the most remote areas, a unique or single dose would make an ideal treatment, as is the case for acoziborole. Its use, together with a rapid diagnostic test, would simplify the process even further. This last matter is rather difficult because HAT symptoms displayed by patients are nonspecific, and accurate tests require skilled personnel. In addition to that, there are still no optimal molecular biomarkers described for an easy-to-use test. Acoziborole is currently undergoing phase II/III trials in the DRC and the Republic of Guinea against the disease caused by the most common strains of the parasite. Remaining challenges include recruiting patients, diagnostics, robust documentation for patient participation, testing close to patient homes, and the needed infrastructure necessary for the trials to take place (De Rycker et al., 2023; Field et al., 2017).

Regarding CD, the aims of DNDi are to develop an oral, age-adapted, shorter-course treatment that is safer and more effective than current options. The organization also aims that the new drug may be used in all regions and for both chronic and acute patients, including pregnant patients (DNDi, 2024). The focus is oriented in the short term toward improved treatment regimens with benznidazole, decreasing the mother-to-child transmission, and designing and implementing “test-and-treat” strategies to reach people living with CD in remote areas of LA, while always having in mind the aim to develop drug candidates to reach at least one Phase III trial.

Thus, together with the Institute Pasteur Korea, the University of Dundee, and Nagasaki University, the identification of a new series of active hits against *T. cruzi*, involving the screening of collections of both synthetic and natural product origin compounds, is being done with the aim of identifying high-quality novel compounds both in chemical structure and mode of action. In parallel, and with partnerships around the globe, advanced leads showed promising efficacy in *in vivo* models. One example is DNDI-6148 (Mowbray et al., 2021), which, however, has been paused pending further studies to determine the potential for reproductive toxicity, as is the situation for benznidazole and nifurtimox, which cannot be used by pregnant women or women who may become pregnant. New proposals for the use of benznidazole regimens have been tested since 2021 (Torrico et al., 2018).

A diversity of treatment options available is essential to overcoming drug resistance. *Leishmania* is an example for which the Wellcome Trust and DNDi have worked together since 2018 with the aim of developing new oral treatments. For example, LXE408 (Nagle et al., 2020), known as a selective inhibitor of the kinetoplastid proteasome since 2020, is now in early development with Novartis at phase 1 trials. It is a derivative of GNF6702 (Khare et al., 2016), whose development was paused due to suboptimal oral absorption. A second proteasome-inhibiting compound, GSK3494245 (Wyllie et al., 2019), showed clinical efficacy in a mouse model of VL, although its phase 1 clinical trial (NCT04504435) was suspended in August 2022 because a participant had an adverse event that met the protocol-defined criteria to stop the trial (eBiomedicine, 2023). As a summary of what has been described, for leishmaniasis, as for CD, there remain many challenges to the development of new drugs that can halt the advance of this disease.

To preserve those compounds that are available and the ones to come, we must develop monitoring tools to detect the existence and prevalence of drug resistance within the population. Diagnostic tests to be developed should be accompanied by a test that can determine the therapeutic outcome of the used drugs and should include the appropriate rules to use them and follow their use closely enough to prevent the prevalence of suboptimal doses of the compounds either because of incorrect use or of their inappropriate availability (Mahopatra, 2014).

**FIGURE 2:**
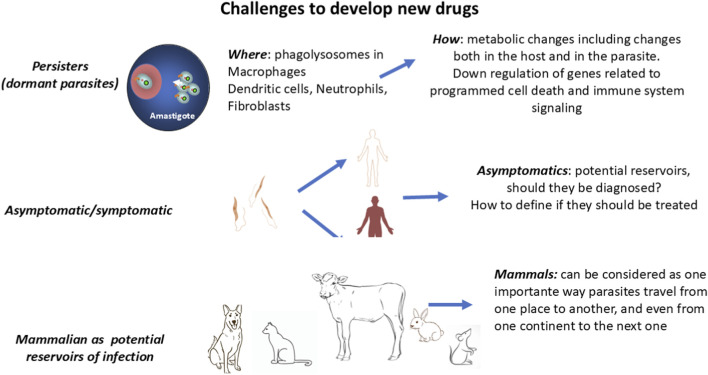
Challenges to developing new drugs against leishmaniasis. Dormant parasites can be defined as a transient population that, due to metabolic changes, does not replicate or replicates at a slower pace, hides in cryptic places, and could be less susceptible to drug treatment. These issues, together with the unknown percentage of infected people who can be considered asymptomatic (or indeterminate in the case of CD) and the potential reservoir role of infected mammals, constitute real challenges to the development of successful medication, which should be addressed by the One Health concept.

Some causes of the challenges involved are depicted in Figure 2 for *Leishmania* parasites; many are also applicable to the trypanosomes. However, some characteristics inherent to *Leishmania* parasites contribute to this situation, as is the case with the existence of emerging hybrids, the dilemma of therapeutic failure vs. emergence of drug resistance, and the existence of aneuploidy, as will be described immediately.

## 7 At the genetic crossroads of *Leishmania*: emerging hybrids reshaping disease patterns

*Leishmania* is such a complex parasite and the associated disease has such a wide spectrum of symptoms that, nowadays, it is common to see, using up-to-date techniques such as multilocus genotyping and whole-genome sequencing, that some so-called “visceral species” may cause cutaneous disease, thus implying that genomic variants are certainly implicated in atypical disease dynamics and that strain genotype, rather than species identity *per se*, determines disease outcome. This is so, even though *Leishmania* species are classified into four subgenera, namely *Leishmania*, *Viannia*, *Mundinia*, and *Sauroleishmania* (Akhoundi et al., 2016), according to their geographic distribution, vector compatibility, and vertebrate host range. Thus, the exploration of their epidemiology must include the investigation and comprehension of their genetic heterogeneity and the drivers that induce in them new variants causing atypical disease, their influence on the evolution of virulence, and the spread of drug resistance. Mechanisms of genetic exchange with distinct potential biological impacts and evolutionary significance have only been demonstrated in the *Leishmania* stages in the sand fly, the host in which these mechanisms are not mutually exclusive and may represent independent, extant processes in natural populations (Ferreira, 2025).

When humans invade the vectors’ ecological niches, the risk of vertebrate host and sand fly coinfection by multiple strains/species that may result in the emergence and spread of hybrid populations is increased. This may lead to atypical variants of public health importance, with outbreak potential and new animal reservoir associations. For example, meiosis-like recombination has been observed in *Leishmania* in sand flies together with recently described self-mating (i.e., intra-clonal sex) and/or backcrossing. Both mechanisms promote genetic diversity. The first one, by following a Mendelian segregation pattern, combines the genomes of both parents (Akopyants et al., 2009; Inbar et al., 2019), and the second can further expand the potential for novel genetic traits (Ferreira and Sacks, 2022; Inbar et al., 2019).

In addition to these methods, non-sexual means can also help in the exchange of parasites’ genetic material. This is the case of *Leishmania* parasites releasing DNA-loaded extracellular vesicles (Douanne et al., 2022) that, when taken up by other parasites, mediate horizontal gene transfer (Soler and Forterre, 2020). Drug-resistance gene amplicons can also be delivered to drug-sensitive parasites through this means (Douanne et al., 2022; Soler and Forterre, 2020).

Finally, it is still poorly understood how the generation of a progeny (>2n) occurs. The hypothesis is that there is a fusion of diploid (2n) *Leishmania* or of haploid (1n) and diploid cells. *In vitro* conditions favor DNA interchange by this route, especially when conditions are imposed that produce DNA stress (Louradour et al., 2022) in these parasites. However, the frequency with which this mechanism occurs is rather low, and it has only been observed in experimental crosses within the sand fly (Akopyants et al., 2009; Inbar et al., 2019). A reverse situation, that is, a reduction of genomic content in experimental conditions, has not been described.

**FIGURE 3:**
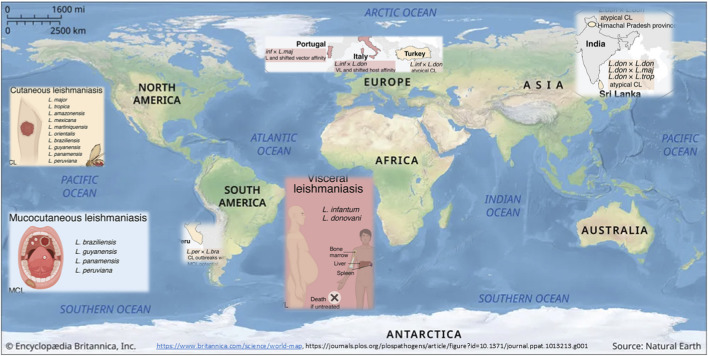
Distribution of visceral and cutaneous leishmaniasis and causative agents, including hybrids that are reshaping the disease patterns [Modified from Ferreira (2025)]. Leishmaniasis is a spectrum of diseases with clinical outcomes that range from CL and MCL to the deadly VL. In this figure, there is a representation of a non-exhaustive list of species typically associated with each form of leishmaniasis. *L.* (*Mundinia*) *martiniquensis* and *L.* (*Mundinia*) *orientalis* that are transmitted by biting midges; all other species listed are transmitted by phlebotomine sand flies. On the world map, examples of natural hybrids exhibiting epidemiological shifts are located in three different regions: South America, the Mediterranean, and South Asia.

Figure 3 illustrates the distribution of VL and CL and causative agents, including hybrids that are reshaping disease patterns. Examples to be mentioned are *L.* (*L.*) *infantum* (VL agent) and *L.* (*L.*) *major* (CL agent), highly divergent natural hybrids that have been isolated from immunocompromised VL patients in Portugal in the late 1990s (Ravel et al., 2006). Such genetic exchange might allow parasites to exploit new vectors or vertebrate hosts, as in this case, suggested by the experimental demonstration that two hybrids generate mature infections in the midgut of an *L.* (*L.*) *major*-exclusive vector, *P. papatasi*, not permissive to *L.* (*L.*) *infantum* strains (Volf et al., 2007).

*Leishmania* isolates from northern Italy were demonstrated to be hybrids from Cyprus, including an *L. infantum–L. donovani* cross lineage with a special affinity for the human vs. the canine host (Bruno et al., 2024). In Turkey, an initially typed *L.* (*L.*) *infantum*, including a cross lineage of *L.* (*L.*) *infantum and* *L.* (*L.*) *donovani,* having, respectively, a special affinity for the human *and* canine hosts, seemed to be causing CL cases in the Çukurova region (Rogers et al., 2014). In Sri Lanka, extensive hybridization and introgression events have shaped atypical disease patterns (Lypaczewski and Matlashewski, 2021). *L.* (*L.*) *donovani* and a smaller population of endemic *L.* (*L.*) *tropica* strains, the former a prototypical VL species and the latter a classical CL species, both cause CL on this island (Silva et al., 2024). Moreover, in Sri Lanka, high genomic heterozygosity in *L. donovani* isolates and multiple cross-species hybrids between *L. donovani* and cutaneous *L. major* and *L. tropica* have been demonstrated, implying a link between genetic exchange and atypical cutaneous disease. Interestingly, although in this case intra- and interspecies hybridization may explain why *L. donovani* infection on the island manifests as a skin-localized disease, a phylogenetically distinct *L.* (*L.*) *donovani* has been described to cause deadly visceral disease in India and an *L.* (*L.*) *donovani* hybrid causes CL without prior VL manifestation only in the Himachal Pradesh province in India (Ferreira, 2025).

Genetic exchange among South American *Leishmania* species has led to frequent hybridization in areas where species coexist sympatrically. This is especially so within the *Viannia* subgenus. For example, *L.* (*V.*) *peruviana* has long been known as the cause of mild cutaneous lesions in the Peruvian Andes, while mucocutaneous disease is due to *L.* (*V.*) *braziliensis*. However, since the 1990s, *L.* (*V.*) *braziliensis*–*L.* (*V.*) *peruviana* hybrids have been associated with severe cutaneous disease outbreaks in Peru, including mucosal lesions (Heeren et al., 2024). This hybrid lineage causes a severe experimental hamster cutaneous disease, including a late and aggressive relapse compared to either parental species (Cortes et al., 2012), which is probably linked to the increased copy number of the surface virulence factor GP63.

## 8 Aneuploidy and adaptation to life cycle stage

Nutrients, pH, and temperature differ enormously between insect vectors and mammalian hosts (Dumetz et al., 2017; Reis-Cunha et al., 2018). This fact directly impacts the parasite’s capacity to display ploidy variation, as has been observed between life cycle stages of *Leishmania*, thus suggesting that *Leishmania’s* adaptation to different hosts may involve aneuploidy. This all means that the parasite, in response to changes in the surrounding environment, is capable of regulating gene expression through aneuploidy, even changing gene transcript levels (Dumetz et al., 2017) between promastigote and amastigote life cycle stages.

Additionally, analysis of *L.* (*L.*) *mexicana* strain RNA-sequences has demonstrated that polysomy may exist even within the amastigote stage. This is the case, for example, with *L.* (*L.*) *mexicana* chromosome 30 (corresponding to chromosome 31 in other *Leishmania* species), which encodes several factors (including transmembrane glycoproteins) whose expression increases in the amastigote compared to the promastigote stage, which aid and are involved in *Leishmania* survival within the mammalian host (de Paiva et al., 2015; Reis-Cunha et al., 2018). Differential somy between promastigote and amastigote life cycle stages has been demonstrated in additional scenarios (Dumetz et al., 2017) with different chromosomes either increasing or decreasing their somy. Determination of which gene on each chromosome is the causative factor of the survival advantage has not been sufficiently evidenced.

## 9 Drug resistance vs*.* therapeutic failure: an additional challenge, using *Leishmania* as an example

The Wisdom Library (https://www.wisdomlib.org/concept/treatment-failure) defines treatment failure as “a situation where medical interventions do not achieve the intended outcomes”. This can manifest in various ways, including the worsening of a condition, the development of drug resistance, or a lack of improvement despite treatment. In the case of leishmaniasis, this concept goes far beyond DR, meaning that these terms are not synonyms. This is so because host and parasite factors conspire to affect outcomes of treated *Leishmania* infections. Factors include drug-, parasite-, and host-related issues, along with coinfections such as HIV. On the other hand, parasite characteristics related to drug resistance include *Leishmania* genome plasticity and parasite molecular modifications that incorporate drug transporters, membrane constitution, and changes in oxidative stress, among others (Ponte-Sucre et al., 2017).

CL is a disease that globally affects millions. Low to middle-income countries are the most frequently affected, and leishmaniasis is almost always found together with other ailments such as malaria, tuberculosis, and HIV. The affected places usually have limited healthcare budgets and rely on poor healthcare infrastructure. These issues, together with a lack of disease management and public health control interventions, positively impact the surge of CL as a potentially uncontrollable disease in most continents. To contain CL incidence and morbidity, there is an urgent need for intensified preventive research programs to guide toward improved vector control, vaccines, and diagnostics (de Vries and Schallig, 2022). Additionally, there is a lack of effective new drugs, because investment in their development for these types of diseases is usually very low, as the economic return is not profitable for pharmaceutical companies. Current molecular techniques allow easy *Leishmania* species identification, a fact that could enable rational therapy management. However, CL treatment guidelines used currently are mostly empirical and lack the required sound evidence, usually because of improperly designed and ill-conducted trials, thus highlighting the urgent need for large, standardized, state-of-the-art trials to evaluate treatments and for updating the way new compounds are identified.

The *Leishmania* parasite promastigote life cycle stage exhibits disparity in gene copy numbers compared to the amastigote life cycle stage, suggesting that variation in copy number may contribute to parasite adaptation to the ever-challenging environment they face, synonymous with the survival advantage of *Leishmania* (Moody, 2021). Wild-type strains and drug-resistant *Leishmania* mutants display differential patterns of chromosomal somy, the “mosaic aneuploidy,” which can be a key issue in drug-resistance development (Moncada Diaz et al., 2024; Sterkers et al., 2012).

This mosaic aneuploidy is a guarantee for strain genetic heterogeneity, even when facing a genetically homozygous cell population (Sterkers et al., 2014). Elimination of deleterious mutations while simultaneously allowing the retention of beneficial mutations is a passport for the guarantee of removing and retaining (down- or up-regulating) the expression of a deleterious mutation or a beneficial mutation. Aneuploidy allows the parasite to use the advantages possessed by both haploid and diploid genomes (Sterkers et al., 2012. “Oscillating” between haploid and diploid genomes is a clever strategy in which mutations in haploid genomes would potentially have immediate phenotypic effects, for example, promoting survival in situations of rapidly changing environments when beneficial mutations appear and are selected and mitigation of the effects of deleterious mutations in diploid genomes is more successful in protecting against potentially harmful mutations.

Of course, such a strategy implies that survival and aneuploidy connected to gene expression alteration infers advantages for drug-resistance development and adaptation to the environment (Dumetz et al., 2018; Ubeda et al., 2008; Vanaerschot et al., 2014). For example, it has been demonstrated that ploidy differed between wild-type and methotrexate (MTX)-drug-resistant *Leishmania major* strains (Ubeda et al., 2008). Patterns that differed between both strains included chromosomes 22 and 28 that displayed polysomy in the drug-resistant strain, whereas chromosomes 11 and 12 displayed monosomy in the wild-type strain, suggesting the selective pressure induced by drug treatment upon *Leishmania* and indicating a role for ploidy in the drug-resistance development.

Remarkable genomic plasticity expressed by *Leishmania* reinforces their adaptability to drug pressure. Aneuploidy, copy number variations, and single nucleotide polymorphisms that collectively facilitate rapid adaptation to therapeutic interventions characterize the significant instability of the parasite genome (Laffitte et al., 2016) and reinforce the occurrence of multiple processes in *Leishmania* involving drug-resistance development through various mechanisms, including altered drug transport, target modification, and enhanced metabolic detoxification pathways (Ponte-Sucre et al., 2017). This genomic instability means an evolutionary gain that permits the selection of resistant populations at a rapid pace when under drug pressure, which results in extensive chromosomal amplifications and deletions occurring in response to drug exposure, signifying frequent copy number alterations (Leprohon et al., 2014). Altered expression of genes involved in stress response, metabolism, and drug transport forms the genetic foundation for resistance development (Ubeda et al., 2008). Genomic plasticity is a key element for resistance, although how evolution traced the trajectory of the effect of drug pressure to impact resistant *Leishmania* is an open question that must still be elucidated (Santi and Murta, 2022).

Additionally, as host-parasite interaction is another key element in this process, host immune modulation and its molecular mechanisms are paramount in drug resistance and require further exploration to elucidate whether resistant parasites alter host immune responses (Costa-da-Silva et al., 2022). Many different mechanisms guarantee the emergence of drug resistance (Afrin et al., 2019) and cross-resistance to different drug classes, such as miltefosine and amphotericin B (Zhang et al., 2025). Finally, to confirm the genomic and transcriptomic analyses, gene knockout or overexpression studies are needed to validate the functional attributes of those genetic and phenotypic changes (Bharadava et al., 2024).

**FIGURE 4:**
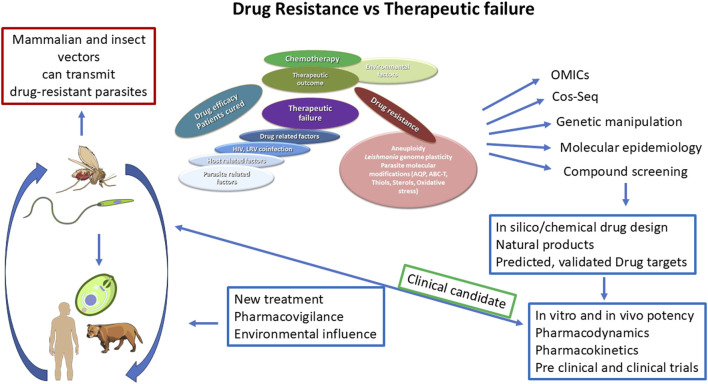
Fate of therapeutic failure vs*.* drug resistance. The figure illustrates the complexity of the fate of therapeutic failure and that of drug resistance in *Leishmania* parasites and the different steps to be taken to comprehend the mechanisms involved.

Thus, the fate of therapeutic failure and that of drug resistance in *Leishmania* parasites is very complex (see Figure 4), and the different steps to be taken to comprehend the mechanisms involved require an exhaustive search of molecular, functional, and pharmacological trends that are intertwined to produce an outcome that may be unique in each case.

The comprehension of the mechanisms that lead toward drug resistance in *Leishmania* parasites is a fundamental step for the progress of antileishmanial treatments that could evade or target these drug-resistance mechanisms. Mechanisms responsible for *Leishmania* adaptation toward drug pressure allow for the development of treatments targeting these mechanisms. Tools that may be used include the sequencing of the *Leishmania* genome and the subsequent identification of differential gene expression. Recently published reviews cover this complex topic very well, and we invite the readers to deepen their knowledge about *Leishmania* drug resistance by reading them (Bharadava et al., 2024; Bhusal et al., 2025; Moncada Diaz et al., 2024). The data discussed in these articles may serve as guidance to understand drug resistance, differentiate it from treatment failure, and help to direct decisions toward how to select the right choice of drug. For example, fluorescence *in situ* hybridization (FISH) and whole-genome sequencing are useful tools for evidencing *Leishmania* heterogeneity in ploidy expression (Ferreira and Sacks, 2022), a character that has been observed between drug-resistant *Leishmania* mutants and wild-type strains. These data suggest that there is a role for mosaic aneuploidy in the surge of drug resistance. To advance this knowledge, both reverse genetic analysis and forward genetic studies are urgently needed.

Development of therapeutic strategies tailored to the life cycle stage may include identification of differential gene expression between the promastigote and amastigote forms of the *Leishmania* parasites, strategies that may be helpful for better controlling the disease, for example, by attacking the promastigote stage within the vector (González-García et al., 2025).

What is clear is that treatment failure is a disturbing challenge in the management of leishmaniasis. This is so because chemotherapy is a central step for disease control, and the magnitude and complexity of the challenge include the full understanding of host immunity, drug pharmacokinetics and susceptibility, and the tolerance and resistance profile of the causative *Leishmania* parasite, which all play a role (Mohapatra, 2014). Thus, acquired drug resistance against the pentavalent antimonials is present in diverse places such as South Asia and East Africa, and even in Latin America (Bhusal et al., 2025; Wijnant et al., 2022). This fact has promoted the use of alternative drugs such as miltefosine and liposomal amphotericin B. Even more, the emergence of resistance in places where high prevalence of HIV/VL coinfection exists implies a further risk for anthroponotic transmission of resistant parasites between patients, meaning that close diagnosis and surveillance are needed, although the lack of validated genotypic and phenotypic assays impairs an appropriate surveillance system (Wijnant et al., 2022).

More than two dozen drugs or combinations have been used in human leishmaniasis (Fernández-García et al., 2025; González-Fajardo et al., 2015; Goswami et al., 2020). In 2004, the WHO-OMS (2004) concluded that the most promising drugs were amphotericin B in its liposomal presentation, miltefosine, and paromomycin (Singh et al., 2023). However, the paromomycin short time-to-resistance in the lab, and subtherapeutic exposure risk to miltefosine and liposomal amphotericin B in patients due to their price, means of administration, and long half-lives sound a note of caution (Olías-Molero et al., 2021).

Cheaper and easier-to-use whole-genome sequencing methods, along with formal rules to quantify and interpret the results of antileishmanial susceptibility testing, must be introduced into scenarios for developing improved therapeutic interventions to identify phenotypic expressions of mutations associated with parasite resistance. Although new drugs (as we have seen) are currently under (pre)clinical development, the ideal therapy should combine these compounds with host-directed therapies or be used in combination therapy with carefully selected partner drugs to optimize and safeguard their future efficacy.

Research on potential new drugs and targets has been very active in recent decades (Croft and Coombs, 2003; Monzote, 2011; Sundar and Singh, 2018, and references within), including by the DNDi and the appropriate institutes/industries to whom they outsource the research. Thus, different compounds and chemical families have been identified as potential hits and leads, and some have been tested in clinical trials (González Matos et al., 2024).

Drug discovery and development of antileishmanial drugs follow the same pattern as any other drug, which includes going from identification of hits, either from natural sources, chemical libraries (from pharmaceutical companies or public access, or small chemical collections from academia), and optimization to leads, and from drug candidates to clinical trials. It relies on repurposing molecules used for the treatment of other diseases. The development involves toxicity tests, pharmaceutical chemistry, and determination of molecule druggability, adsorption, targeted distribution, metabolism, and excretion of the molecule and other pharmacokinetic parameters. The process is long, expensive, and, despite all the steps included, does not guarantee that the selected candidate will not fail at the clinical stage (Olías-Molero et al., 2021). Thus, in addition to the scientific threads that may guide drug development, a note of caution about the role of academia should be mentioned.

## 10 A note of caution about the role of academia

Research in academia includes publishing results. However, most published articles focus on potential antileishmanial molecules only from the basic point of view; that is, *in silico* design, chemical synthesis, molecular docking, and *in vitro* and *ex vivo* antileishmanial activity assays (mainly in the promastigote stage) (De Rycker et al., 2013), which have a lower predictivity on antileishmanial human disease and even less on *in vivo* settings. Even now, the literature has a high proportion of studies based only on predictive *in silico* analysis, not even suggesting the next step toward *in vitro*, *ex vivo*, or *in vivo* testing. Academic research groups seldom have the appropriate size, expertise, funding, and availability to try more than the basic steps toward the molecular characterization and experiments into the preclinical drug pipeline research: medicinal chemistry/chemical pharmacology is not sufficient to describe the whole set of assays that must be done to compete, for example, with systematized companies. This goes from molecule synthesis up to determination of toxicity for mammalian cells and tissues, through *in vitro* and *in vivo* efficacy, mechanism of action, pharmacokinetics, and pharmacodynamics.

Within this long road to patient care, challenges in new drug discovery, mainly to avoid/reduce failure in the translation process, should be taken into consideration and analyzed in all areas, including the main one of our interest: the development of antiparasitic drugs. Potential causes of attrition include many matters, from structural to operational, such as the scarce reproducibility of preclinical findings (Seyhan, 2019) and that go from the preferential funding of basic knowledge to the strategical and administrative divide between preclinical and clinical investigation (Fernandez-Moure, 2016), including the use of only partially validated targets (Bhattacharya et al., 2020; Seyhan, 2019), or inadequate animal models. One good rule that is usually not followed is to drop a compound early enough not to harm or introduce confusion in the data if it does not yield sufficiently reliable results in *in vitro* testing. This is a sound philosophy to facilitate the task of providing solutions (Fernandez-Moure, 2016) and challenging ourselves through the established steps and procedures because our aim is to translate from lab bench to bedside (Seyhan, 2019) at the fastest pace possible.

As has been pointed out by the group of Olías-Molero et al. (2021), driving/braking forces of the process of attaining a new antileishmanial drug can profit from very concrete actions such as goal-oriented collaborative work, selection of adequate surrogate animal models, and reconsideration of target product profile (TPP) requirements, among other factors that also should be considered. Their point of view that we share is that these three aspects play fundamental roles when exploring new chemical entities and repurposed/repositioned drugs or drug combinations, beyond the laboratory, to effectively control the severity and extension of leishmaniasis.

The need for collaborative research activity sharing objectives relates to breaking walls between groups to share knowledge to make the experience more profitable in identifying new antileishmanial drugs or drug combinations. Regarding animal models, their point of view goes toward the fact that, regardless of the final recipient of a drug, humans or domestic animals, experimentation is required. Assays performed in animals are always a must as a prior step toward preclinical and clinical assays (Campbell et al., 2016), as has been deeply discussed by the European Animal Research Organization (EARA) (www.eara.eu; accessed on 26 November 2021). In the case of antiparasitic drugs, this necessary step decreases attrition rates usually observed for compounds in phase II involving human and veterinary patients (Pink et al., 2005), although the animal models used are usually far from ideal in the case of human patients. However, there is no ideal model available for antiparasitic drugs intended for humans and the fact that these new compounds should (ideally) be compared to established ones makes the tests design rather complicated without a clear-cut result at the end (Alvar et al., 2006; de Freitas-Junior et al., 2012), even without considering the need for an oral compound that ideally does not need medical surveillance but that also has very serious limitations both from the pharmaceutical (more complex to develop) and population (for example, price) point of view. This means that the protagonism of the pharma industry is a key factor for being successful, and their commitment is fundamental for crystallizing translational research.

## 11 Role of the pharma industry

Turning to the pharma industry, we must recognize that molecular biology methods (e.g., genomics, proteomics, transcriptomics, and other–omics) and selection of hits/leads/candidates based on the target-oriented and mechanism of action paradigms (Jain and Jain, 2018) have been included for the past 30–40 years as technical approaches. Together with high-throughput screening method systems (HTSs), these methods have the aim of reducing the previous high attrition rates seen with using natural products as the source of leading compounds and then chemically modifying them in the goal to encourage translation from *in vitro* toward *in vivo*, preclinical, and clinical candidates (Bhattacharjee et al., 2024; Saldivia et al., 2024). However, this novel approach, especially HTSs, has fundamental limitations when using, for example, intracellular stages of *Leishmania* or *Trypanosoma cruzi* (Saldivia et al., 2024; Bhattacharjee et al., 2024). This has resulted in the reality that, to date, high-throughput screening and target-based selection have not reduced attrition rates and have not provided the hits and leads that were expected in any therapeutic field, including antileishmanial treatments. This is a generalized issue that affects the pharma companies, basic scientists, and those who are near the patients or the affected animals, such as medical doctors and veterinarians. Even more, most drugs employed against *Leishmania* infections constitute repurposed drugs and treatments and are not new chemical entities.

One of the main challenges is going beyond the achieved academic results. Only very few new chemical entities have been identified for leishmaniases, and the evidence obtained from field conditions normally is not conclusive (Geary et al., 2009; Scanell et al., 2012; Woods et al., 2011). Even *in silico* and bioinformatic approaches have followed a similar destiny. We await what artificial intelligence, predictive platforms (Woods et al., 2011), and automated drug discovery (Schneider, 2018; Singh et al., 2024; Woods and Williams, 2007) will provide.

Other complicating aspects to consider are the complete separation between research and development and financial departments within the companies (Cuatrecasas, 2006), the higher stringency of present requirements by regulatory agencies together with inadequacy of patent protection (Grabowski et al., 2015; Santos Rutschman, 2021; Trouiller et al., 2002; Wenig et al., 2018), and the contraction in the number of operating companies due to globalization (Akinori et al., 2023).

This means that there are no ideal treatments and, although we must appreciate that decisions from the pharma industries can be impacted by the lack of significant revenues from antileishmanial drugs and drugs for other NTDs such as HAT, CD, schistosomiasis, and geohelminths, it cannot be considered the factor most responsible for the low investment by pharma companies. Therefore, the absence of new chemical entities (Martin-Plaza and Chatelain, 2015; Trouiller et al., 2002) is a factor that must be evaluated from other points of view.

Additional issues to be addressed include the fact that these diseases affect people who live in poor countries with endemic disease (Alvar et al., 2006), in scenarios in which the health of an individual or a human community is beyond the treatment of a single disease and medical supervision and the need (sometimes unrealistic) for one drug that can fight all types of disease. This implies that the role played by the policymakers is key to achieving success.

## 12 Role of adequate policies

Addressing political issues, such as the United Nations’ call to action on 21 September 2023 for “Universal Health Coverage (IFPMA, 2023): Moving Together to Build a Healthier World,” is essential to accelerating progress toward achieving universal health coverage by 2030. Similarly, prioritizing the global agenda to end NTDs reflects a commitment to upholding the human rights of people living in poverty, hunger, and precarious conditions, often without access to clean water, sanitation, or education. Tackling these challenges is both a matter of health and a crucial step toward reducing inequities worldwide (World Health Organization, 2023). One phrase unites these issues. The One Health approach to consolidate stronger collaboration in all areas involved, that is, health, water, sanitation and hygiene, education, and food safety in an ethical environment of a public health framework that faces challenging issues due to different themes such as serious adverse reactions associated with preventive chemotherapy, centralization of decision making, or adequate policies for school-based deworming (Lancet series on Lancet, 2023; Vasan et al., 2017; Ung et al., 2021). The One Health approach means taking into consideration the holistic atmosphere that should surround the conventional model of zoonotic disease control, to surpass the paradigm and consider that we are all immersed in a world that is, as Alexander von Humboldt called it, “Nature Gemälde,” in which the interactions of human and animal health systems and the surrounding environment, including the socio-economic context, are a must. Otherwise, the goal to be achieved, that is, “Universal Health Coverage: Moving Together to Build a Healthier World” (World Health Organization, 2023), will be only a nice dream and not a concrete goal that, if not achieved, could lead to the extinction of human beings. This is why there is an urgent need for political will and innovative scientific strategies, both involved in the context that we are living, to be successful, from the physical but also from the psychological (stigma and mental health) point of view. A broader scope, to include provision of rehabilitation and linkages to mental health support and tackling stigma through demystifying NTDs, is required to promote the health of affected people (Kuper, 2021; Laing, 2023; Laing et al., 2021; Malecela and Ducker, 2021).

These threats to be considered can be divided into three areas: 1) emerging infectious diseases, 2) exploding population without improved health, and 3) erosion of humanity and leadership if we turn our backs on the health problems of the growing majority of people on our planet. These threats need a quick and firm response to gather real-time information and build collaborative networks aimed at enhancing surveillance to develop high-priority medical countermeasures to prevent and control emerging tropical diseases. Grand challenges thus remain in the laboratory, our daily work, in the hospitals, in the field, in the community, and in many other places, to reduce the impact of emerging tropical diseases, let’s work on this together (Gutiérrez-Ocampo et al., 2021).

## 13 Role of the One Health approach

In the 19th century, Rudolf Virchow used the term “One Medicine” and demanded the collaboration between veterinarians and medical doctors (Prata et al., 2022). Nowadays, it implies interaction among professionals of different disciplines involved in human health (doctors, nurses, public health practitioners, and epidemiologists), animal health (veterinarians, paraprofessionals, and agricultural workers), and environmental health (ecologists and wildlife experts) to communicate, collaborate on, and coordinate activities (CDC- Atlanta, 2025). Other relevant players in the One Health approach could include law enforcement, policymakers, agriculture, communities, and even pet owners. No one person, organization, or sector can address issues at the animal-human-environment interface alone (CDC- Atlanta, 2025).

Thus, the concept of having a common health approach, or the One Health notion, is already approximately two centuries old. By no means does this imply that a collaboration between both professions has lasted for a long time (Woods and Bresalier, 2014) and, even more, the 20th century was witness to a separation between the two careers and fields of knowledge that ended up in the isolation of each (Woods and Bresalier, 2014). The 21st century, a time of huge interconnectivity, globalization, and accelerated expansion of cultures throughout the world, demands that veterinarians and medical doctors work together to plan and execute programs to impact communities regarding population health, based on scientific research on medical topics that affect all types of communities and populations.

The main reason for this approach is the fact that we, the world, constitute a net, a web, due to the deep interconnection that exists between animals, environment, and humans (World Veterinary Association, https://worldvet.org/policies/wva-position-statement-on-one-health/, Accessed November 22, 2025). The balance among these three protagonists is a key factor in maintaining planetary health, well-being, and global health (Calistri et al., 2013). We are first-line observers and protagonists of what is happening: 2 years ago, we reached 8,000 million people living in the world (World Meters, 2025). Due to the classical view of humans being the sovereign of the world, we are ruining it, losing biodiversity, and promoting extreme climate changes and fragmentation of our habitat, all of which trigger the appearance of emerging and reemerging diseases.

On the other hand, ancient events impose current pathogenic relationships that burden society. Ancient genes, exposed to more than a billion years of natural, microbial, and chemical warfare, play a role in antibiotic resistance today. Humans, poorly adapted to their living conditions, promote an imbalance of host-commensal relationships that become parasitic, ancient human microbiome relationships that, once disrupted, become dysbiotic, resulting in an overreaction of host immunity. How these issues happen remains a mystery, but we see them around us. These issues then guide us to pivotal questions such as “What ancient and widespread genes are poised for novel antimicrobial resistance?”, “Why is one host relationship commensal and another pathogenic?”, and “How do the ecosystems in and surrounding our bodies change?” (Lewis et al., 2023).

Due to this chaotic panorama, many international organizations, among them the Pan American Health Organization (PAHO), have promoted the One Health approach for several decades; PAHO included the concept in its official agenda in 2021 and defined it as a “*comprehensive approach for addressing health threats (endemic diseases of zoonotic and vector-borne origin, emerging, and reemerging infectious diseases of zoonotic origin, antimicrobial resistance, and food safety) at the human–animal–environment interface*” (Aggarwal and Ramachandran, 2020). For this organization, the will and action should focus on working in collaboration with the native and minority populations of LA and learning what these ancient cultures have to teach, as well as teaching them about the advances that may positively impact the local health systems, thus implementing an effort to promote dignity and diversity, as well as a positive balance toward human-animal-plant-ecosystem harmony (Pettan-Brewer et al., 2021).

In doing so, and in this case for diseases caused by Trypanosomatidae and the increased awareness of the different clinical manifestations caused by those diseases, it looks like their diagnoses and treatment must undergo major adaptation according to the realistic challenge confronted. These practices must be directed both toward infected humans and to other vertebrate hosts (reservoirs) involved in the transmission of zoonotic diseases due to their role in the transmission to humans of these diseases.

In addition to the need for techniques to ensure faster and accurate diagnosis, the improvement of drug screening tools should be directed to the description and validation of potential drug targets and molecules with adequate and specific antiparasitic activity. To complete this task, we “must ask the parasite”, meaning we must increase our knowledge (identification and comprehension) of parasite biology. This approach fits within the concept of One Health, defined as “the collaborative effort of multiple disciplines to obtain optimal health for people, animals, and our environment” (Behravesh et al., 2022).

An accurate, reliable, on-time diagnostic testing that is easy to implement even by minimally trained individuals to detect and characterize the pathogen within laboratory settings appears to be a key factor to ensure intersectoral integration, control, and elimination of zoonoses (Bidaisee and Macpherson, 2014), including those caused by Trypanosomatidae. All diseases caused by Trypanosomatidae, especially those that are lethal if not treated, challenge us to develop accurate diagnosis as an initial step for faster and more accurate detection of parasitic diseases, which leads to the advancement in treatment and drug discovery (Lewis, 2020; Quaresma et al., 2023; Wenig et al., 2018).

It is impossible to separate human health from the health of the natural world. The scientific potential of a One Health approach is unlimited, exciting, and necessary for the health of life on our planet (Erkyihun and Alemayehu, 2022; Gittleman, 2024).

## 14 Translational research in Trypanosomatidae in a One Health, One World environment: a view from pharmacology

As we have discussed, adequate policies toward universal access to high-quality diagnostics and medicines remain essential to make surveillance successful and delay the unavoidable surge of drug resistance (Hotez et al., 2016; Wijnant et al., 2022).

WHO/PAHO guidelines favor local treatments such as thermotherapy or intralesional antimonials (SbV) for uncomplicated CL; however, most patients in endemic regions are still treated with systemic therapies. The reasons abound, including local protocol misalignment, limited access to new technologies, patients not having the lesions in anatomical areas other than the face or near the joints, lesions being smaller <3 cm in diameter, and presenting a single lesion. These facts constitute challenges for rural patients, especially in endemic areas, with limited access to medical facilities and compromising treatment adherence and clinical follow-up, thus increasing treatment failure.

**FIGURE 5:**
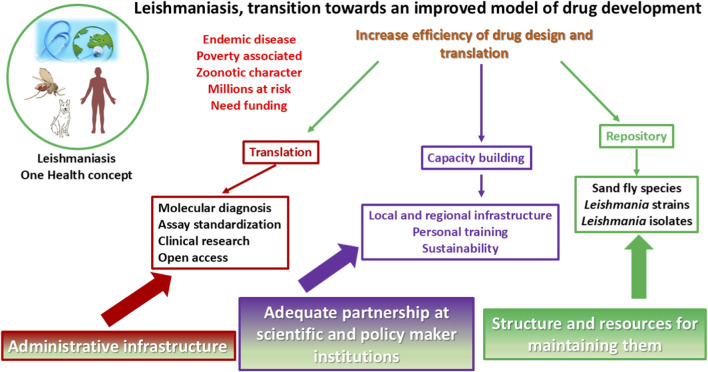
Road of transition toward *Leishmania* parasite drug development. The figure illustrates the steps to be taken from different areas to forward the translation of developed chemical entities into medications against the different forms of leishmaniasis using the One Health approach.

Figure 5 presents a short summary of the steps that should be taken from different areas, as an example, to forward the translation of developed chemical entities into medications against the different forms of leishmaniasis using the One Health approach. A brief description is made herein to emphasize the importance of many of those steps.

For example, in pharmacological–pharmaceutical terms, simple measurements to be taken should yield a lower rate of attrition and increase the potential for success (Olías-Molero et al., 2021; Scannell et al., 2012). These include (1) use of the adequate parasite stage for *in vitro* assays (mammalian intracellular amastigotes), together with the validation of adequate high-throughput screening systems and phenotypic screening that may reduce the selection of irrelevant targets. (2) Use of EC_90_ rather than EC_50_ as a stringent value for *in vitro* and *ex vivo* tests to determine the anti-*Leishmania* activity of a molecule. These two steps would mean the combination of higher stringency and validated experimental design and methods that would allow for more predictive checkpoints. (3) Inclusion of stringent methods for safety and toxicity in early phases of experimentation, including an appropriate description of the selectivity index for the intracellular amastigotes over host cells, as well as toxicity in *in vivo* standard laboratory models, even before testing the efficacy *in vivo*, and (4) performing adequate and broad pharmacokinetics/pharmacodynamics evaluation of molecules, with different administration routes, initially *in silico* absorption, distribution, metabolism, and excretion (ADME) predictions before doing them in standard animal models and, even better, in an advanced preclinical species, to determine major pharmacological parameters (AUC, bioavailability, half-life, excretion rate, and biodistribution of the molecule) before *in vivo* testing*.* This will avoid the loss of time and money and secure scientific or ethical justification, especially toward the *in vivo* preclinical trials (which may include dogs for leishmaniasis) that share both being a preclinical model for human VL and a natural host for zoonotic-VL that nowadays receive the same drugs as humans.

(5) Fundamentally, perform transparent animal trials that include the treatment itself as well as the supportive therapy, for example, in the case of VL, required to restore the physiological status of the patient. The fact that this implies, in most instances, a personalized therapy varying from patient to patient should be taken into consideration when analyzing the data from clinical trials. This issue will increase the validity of any evaluation/comparison of candidate drugs and will improve the actual value of the research carried out. (6) Increase the use of network design and flexible structure, including collaborative work, and avoid the linear pipeline with clear goal-oriented leadership. This should include open access to data repositories to ensure a more precise assessment of the results’ value, to attack together the challenges and find solutions to them. Surely, after so many years with insignificant or very limited success, a new approach to the prevailing practices in drug development designs is needed.

(7) Thoroughly evaluate the *in silico* analyses performed and the implications they have for the development of new compounds. *In silico* computational chemistry data with no hints of *in vitro* or experimental data can produce more difficulties than advantages. The main reason is that the disparity between computational estimates and *in vitro*/*in vivo* studies may result in data that cycle within a never-ending loop, leading to the current situation of enormous efforts yielding no advance in new compounds against the parasite to treat the diseases. (8) Finally, reinforce the role played by pharmacogenomics and “-omics” approaches by real analysis of data in the *in vivo* field.

Without a doubt, the contribution these techniques may make to enhance treatment personalization and improve outcomes, for example, identifying biomarkers for treatment response and toxicity prediction, understanding resistance mechanisms at the molecular level, and repositioning existing drugs to identify several candidates with antileishmanial activity, may be enormous. However, only translating these data into real analysis will give the final positive answer needed.

Thus, while considerable progress has been made in the diagnosis, prevention, and treatment of leishmaniasis, significant challenges remain (Abbasi, 2025). The prevailing model to develop new antileishmanial drugs, although successful at the basic academic step, has demonstrated a very low efficiency, resulting in high compound attrition. Reverting this scarce effective progress, and the additional need of making drug research and development a sustainable activity (Hendrickx et al., 2018; Preziosi, 2004) impose an increased interaction between academia, public institutions, charities, and industries to progress in the numbers in translational medicine (Seyhan, 2019) to reach the patients. It is the challenge we are facing. The question is not whether we (the world, humanity) have resources or motivation to challenge the future, but whether we will survive if we decide not to make this effort (Worldvet, 2025).
